# Interrelation between Patient Satisfaction and Patient-Provider Communication in Diabetes Management

**DOI:** 10.1155/2014/372671

**Published:** 2014-12-28

**Authors:** Ayse Basak Cinar, Lone Schou

**Affiliations:** ^1^Institute of Odontology, University of Copenhagen, Section 1, Norre Alle 20, 2200 Copenhagen, Denmark; ^2^Department of Global Oral Public Health, Institute of Odontology, University of Copenhagen, 2200 Copenhagen, Denmark

## Abstract

The present study aims to assess how patient satisfaction with medical provider-patient communication can affect oral health, diabetes, and psychobehavioural measures among type 2 diabetes (T2DM) patients. It is part of a prospective intervention study among randomly selected T2DM patients, in Turkey. The data analyzed were Community Periodontal Need Index (CPI), HbA1c, patient satisfaction with communication, and psychobehavioural variables. Data was collected initially and at the end of the intervention. The participants were allocated to either health coaching (HC) or health education (HE). At baseline, there were no statistical differences between the HC and the HE groups on any of the measures (*P* > 0.05). Patients in both the HC and the HE groups had low satisfaction with communication. At postintervention, the increase in patient satisfaction with communication in the HC group was significantly higher than that in the HE group (*P* = 0.001). Principal component analysis revealed that patient satisfaction with communication shared the same cluster with clinical measures (CPI and HbA1c) and quality of life in the HC group. In conclusion, the present study showed, to our knowledge for the first time, that overall patient satisfaction with medical care provider-patient communication, empowered by HC approach, was interrelated with well-being of T2DM patients, in terms of psychobehavioural and clinical measures.

## 1. Introduction

The burden of diabetes is increasing globally, particularly in developing countries, and it is expected at least to have been doubled by 2030, where 694 million people are expected to suffer from diabetes [1]. The WHO projects that diabetes will be the 7th leading cause of death in 2030 [2]. Type 2 diabetes (T2DM) comprises 90% of people with diabetes around the world [3] and is largely the result of poor lifestyles.

Patients with periodontal disease are more likely to have T2DM [4–6]; they have a higher risk for further complications such as cardiovascular problems [7, 8] and mortality at early age [9]. An effective prevention method for initiation/progression of periodontal diseases is daily toothbrushing [10], which is part of personal lifestyle. A good quality of patient-provider communication can positively affect the adoption of daily toothbrushing habits because patients value having a supportive and caring dentist and a dedicated dental team. Research has shown that the experience of having a dedicated, supportive, and caring dentist helped patients to take control of their own oral health [11]. However, in the literature, it is not clear how patient-provider communication can influence self-care practices, such as toothbrushing.

The majority of T2DM management is conducted by the patient [12] and diabetes is considered as one of the most psychologically and behaviourally demanding chronic diseases [13]. Many T2DM patients find themselves unable to follow recommended medical regimes and lifestyles (a healthy diet, regular physical exercise, twice daily toothbrushing, and no smoking), which makes them more prone to T2DM-related complications and poor oral health, leading to a poor quality of life [12, 14, 15]. Satisfaction with the patient-provider communication is one of the key elements of adherence to medical regimes [16] and diabetes outcomes [17].

Effective diabetes management requires effective communication between patient and provider, where medical professionals address psychosocial issues and are concerned about the expectations and needs of the patients. As professional diabetes care addresses multidisciplinary team approach (physician, dietician, psychologist, etc.), patients' satisfaction with communication refers to overall satisfaction with the multidisciplinary team of diabetes care. That may have a significant impact on their adherence to medical regimes and adoption of healthy lifestyles. However, the interrelation between patient overall satisfaction with a multidisciplinary team and diabetes management has been a neglected issue. The present study aims to assess how overall patient satisfaction with medical provider- (dentist- and physician-) patient communication can affect oral health, diabetes, and psychobehavioural measures among T2DM patients.

## 2. Material and Methods

The present study is part of a prospective intervention study among T2DM patients (*n* = 186), randomly selected from the outpatient clinics of two hospitals in Istanbul, Turkey. The power and sample size has been explained previously [18–20]. Eligibility criteria were (1) confirmed type II diabetes, (2) 30–65-year-olds with at least 4 functional teeth, and (3) no psychological treatment and hospitalization.

Ethical approval and written permission to conduct the study were granted by the Ministry of Health. Information regarding HbA1c was taken from the latest medical records at the hospital, while the patients were invited for periodontal clinical examination. Of the patients participating, 96% (baseline visit, *n* = 179; final visit, *n* = 178) attended the clinical examinations. Of 186 participants, the dropout was 7 patients (4%) and the corresponding figure for the participants who did not regularly participate in all sessions was 24 (13%).

Back translations to and from Turkish were performed for the health behavior questionnaires by two native speakers to ensure comparability with the original forms in English.

The data in the present study were extracted from the self-assessed questionnaires, clinical measurements, and the medical records taken at baseline and at the end of follow-up. At the baseline visit, participants provided informed consent and filled out questionnaires (including demographic background and psychosocial and behavioural variables). The last current medical reports (glycated haemoglobin expressed as the percentage of haemoglobin that is exposed to glucose (HbA1c), HDL, LDL, and triglyceride) were drawn from the hospital. Subsequently all participants were invited for baseline oral examination which was conducted by two calibrated examiners. Examiners were blind to group assignments.

Following the baseline oral examinations, patients were randomly assigned to either the health coaching (HC) or the health education (HE) groups by means of a block table of random numbers (block randomization by physician). The procedure and randomization were explained in detail previously [18–20]. Following the oral examination, patients entered HC or HE groups as explained above. The study included two phases (10-month initiation and maintenance and a 6-month follow-up). During the 10-month initiation and maintenance, all participants in both groups were invited for free periodontal cleaning and three seminars about oral health and diabetes management. Periodontal cleaning was offered free of charge and it was performed by a dentist (BEA) at a dental polyclinic; there were no oral health care facilities available at the hospitals. In connection with the periodontal cleaning to all participants, standard oral health education was given. All patients were called between one and three times for an appointment. The cleaning included the removal of soft and calcified deposits by an ultrasonic device. At the end of the 6-month follow-up phase, the same outcome measures were collected.

### 2.1. Health Coaching Group

One professional health coach (ABC) provided HC intervention for the participants. ABC had an internationally accredited coaching training. Participants randomized to the HC had a face-to-face session with the coach within 2 weeks of the baseline visit.

HC, described earlier in detail [18], is focused on empowerment of patients for daily health-related practices, compliance to diabetes and oral health-related self-care regimes, building up health-related capacity skills, self-monitoring, and taking responsibility of health and quality of life. Besides, it increasingly targets the awareness of patients for risk factors and possible complications considering diabetes and oral diseases. The primary method is that patients set up the goal and an action plan, focusing on improvement of lifestyle and clinical measures, under the supervision of the coach. Each coaching session is used for subsequent monitoring of patients' progress towards the achievement of the target goal. Preset time frame for face-to-face coaching sessions is 20–60 minutes, determined by needs, expectations, hindrances, and progress of the patient.

HC was supported by educational and motivational brochures, designed for the project. Brochures in the format of written text were given to each patient. Two copies of the brochures were given to the leader and the head nurses of the polyclinics of the hospitals for information and any possible comments. Later on, the brochures were used at “Diabetes School Seminars” by the hospitals.

### 2.2. The Health Education (HE) Group

Participants randomized to the HE group received standard lifestyle advice referring to oral health care practices, diet, and physical exercise. One dentist provided HE intervention. The HE group patients were supported by the same educational brochures as the HC group.

All participants in both the HC and HE groups were supported by 8 text messages at the initiation phases and on special occasions (Ramadan fests, New Year Eve). They also received health promotion samples (toothbrush, toothpaste, sweeteners, and Pilates exercise balls).

### 2.3. Outcome Variables

Oral examination, in particular periodontal examination, was performed by two calibrated clinicians [18–20]. The WHO periodontal probe with a 0.5 mm ball tip was used to determine Community Periodontal Need Index (CPI) by measuring the pocket depth and to detect subgingival calculus and bleeding response. The index teeth at six sextants (17, 16, 11, 26, 27, 47, 46, 31, 36, and 37) were probed and the highest score for each of the six sextants was recorded, in line with WHO recommendations for measurement of CPI [21, 22]. If no index teeth or tooth was present in a sextant qualifying for examination, at least two nonindex remaining teeth in that sextant were examined and the highest score was recorded for each sextant.

Diabetes management was measured in terms of HbA1c. The respective variables were taken from the last health records, provided by either the hospital or the participants.

Patient satisfaction with the medical provider-patient communication was measured by a scale composed of 4 items ranging on a 5-point Likert Scale (“0 = very unsatisfied” to “4 = very satisfied”). The question “How satisfied are you with your … during the last 4 months?” was used to assess the patient satisfaction on the (1) attitude of the physician in general, (2) information/counselling about diabetes management provided by the physician, (3) attitude of the dentist in general, and (4) information/counselling about the role of diabetes in oral health management provided by the dentist. The Cronbach correlation coefficient measures (*α* = 0.701) were good. Further, the split-half internal consistency, applied to test the reliability of the scale, was 0.68 (Equal-Length Spearman-Brown) with the correlation between the two halves being *r* = 0.81.

Questions about self-reported physical activity were taken from an earlier study [23]. Participants answered a multiple choice question like “Please tick the activity that fits you best.” There were four answer possibilities: “(1) read, watch TV or other things in a sitting position, (2) walking, active house work at least four hours per week, (3) jogging, running and other kind of running exercises or hard work in a garden 2-3 hours per week, (4) tough training, competition, sport more than once a week.” Responses were reclassified into three categories by taking the last two categories as one “physically high active” as the responses to each of these categories were not so high. For further analysis, it was dichotomized as “physically inactive” and “physically active.”

### 2.4. Psychobehavioural Measures

Self-reported toothbrushing frequency was taken from an earlier study [14] asking the following: “How often do you brush teeth?” Toothbrushing frequency was recorded on a 5-point Likert Scale (“never = 0, once a week/less = 1, 2–5 times/week = 2, once daily = 3, twice/more daily = 4”). For further analysis, it was dichotomized as “irregular: brushing less than once a day” and “regular: brushing at least once a day”.

The toothbrushing self-efficacy (TBSE) scale [14, 19, 24] was used to assess individual's belief in his/her competency to brush his/her teeth daily across different challenging situations by the question “How sure are you that you can brush your teeth….” TBSE scale consisted of eight items on a five-point Likert Scale (0: “not sure at all” to 5: “absolutely sure”). (For the design and validity-reliability measures of the scale, see [14, 19, 24]).

The quality of life regarding daily diabetes management was modified version of the WHOQOL-Bref [25, 26]. It referred to the physical and psychological domains (Table 1). It included 6 items: 3 physical and 3 psychological well-being-related item (responses on a 5-point Likert Scale; 0: “not at all” to 4: “very extreme amount” for the items 1 and 2 and reverse coding for the items 3–5, and 4: “never” to 0: “always” for the item 6). The validity and the reliability of the scale were tested earlier [25].

### 2.5. Analysis

Statistical analyses were performed using SPSS v.17 (Chicago, Illinois). For assessment of correlation and baseline similarities/differences between the HC and the HE groups, respectively, Spearman rank correlation, independent sample *t*-test and cross-tabulation, and chi square analysis were used. Statistical significance was set at 0.05 for each test.

Factor analysis can be used to hypothesize an underlying construct by the principal component analysis (PCA) approach; thus, it is used to find a few combinations of variables, called components or clusters, which adequately explain the overall observed variation, thereby reducing the complexity of the data, as described in an earlier study [24]. In the present study, factor analysis was applied to the variables by using PCA and Varimax rotation to analyze not the associations but the interrelationships (connection by sharing the common background factors) and common underlying dimensions among patient satisfaction with patient-provider communication, periodontal health, and HbA1c levels and psychobehavioural variables (self-efficacy, quality of life, physical activity, and toothbrushing). These variables were classified into discriminative clusters (latent variables) based on factorial loadings, ranging from highest to lowest values. Loadings below 0.25 were extracted for ease of communication. The clusters are named based on the variable with highest loading. Factors were extracted according to meeting the Kaiser criterion of eigenvalue greater than 1.

## 3. Results

At baseline, there were no statistical differences between the HC and the HE groups on the oral health- and diabetes related measures (*P* > 0.05) (Table 2). Patients in both the HC and the HE groups had low satisfaction with medical provider-patient communication (mean: 6.3 ± 3.7 versus 7.3 ± 3.9  *P* > 0.05).

At postintervention, the increase at satisfaction with the communication in the HC group (mean change = 6.3) was significantly higher than that in the HE group (mean change = 3.1), (*P* = 0.001) (Table 2). The HC group patients, who were highly satisfied with medical provider-patient communication, were more likely to be physically active (94%) than those who were less satisfied (67%) (*P* = 0.001). Such a significant association was not observed in the HE group. In the HC group, those who were physically active were more likely to brush their teeth regularly (92%) compared to those who were physically inactive (64%) (*P* = 0.001). Such a significant association was not observed in the HE group (*P* > 0.05).

Principal component analysis revealed that patient satisfaction with the medical provider-patient communication shared the same cluster with oral health- and diabetes related measures and quality of life among the HC group (Table 3). In the HE group, patient satisfaction with the medical provider-patient communication was interrelated with CPI and HbA1c. Patient satisfaction with provider-patient communication in the HE group was correlated by improvement at self-efficacy (*r*_*s*_ = 0.295) and quality of life (*r*_*s*_ = 0.366) (*P* < 0.05).

## 4. Discussion

We showed that satisfaction of T2DM patients with provider-patient communication is interrelated with health outcomes, determined by HbA1c and CPI in both the HC group and HE group. These findings may add to the growing body of evidence linking patient satisfaction with diabetes related outcomes [17, 27, 28]. The dental literature, scarce in number, suggests that dentist-patient communication directly affects patient satisfaction with the dental treatment [29, 30] or oral health services [31]. However, it is not known how that communication may affect oral health and/or behaviour. The present study shows how overall satisfaction with patient-provider communication is interrelated with both oral health and diabetes.

HC focuses on self-empowerment and it is a patient-focused approach for transformation and maintenance of healthy lifestyles and to improve quality of life. HC helps individuals to transform their cognitive and emotional functioning to adopt positive health behaviours, by setting up personal goals and specific action plans, supported by improvement of self-efficacy. Self-efficacy focuses on one's confidence to perform a given behaviour [32]. Several studies have documented associations between self-efficacy and diabetes self-care [33–35]. Increased self-efficacy and thereby better diabetes management are related with engagement in shared decision making for medical care/treatment and better quality of communication between patient and provider [36]. In the present study, in the HC group, satisfaction with patient-provider communication, oral health and HbA1c, self-efficacy, and quality of life formed a single cluster. That may reveal patients whose self-efficacy improved through coaching intervention took decisions and acted on to improve their oral health and diabetes and thereby improved their quality of life. Increased awareness and self-confidence in their capabilities to improve their quality of life may lead these patients to engage actively in medical consultations/visits. Moreover, patients who take a more active role often are more satisfied with care and more committed to treatment plans, have a better understanding of treatment options, and experience greater improvement in health than do more passive patients [37]. That may also be an explanatory factor for why the patients in the HE group did not have any improvements in the clinical measures. Those patients had less improvement in the self-efficacy; thereby, they most probably did not take an active role in decision making during consultations which may lead to less satisfaction with the communication.

T2DM patients who discuss their treatment goals and management strategies with their physicians tend to have better clinical outcomes than those who do not [38, 39]. The individually tailored and goal oriented programs, particularly based on improving self-efficacy, have been more efficacious in improving periodontal health compared to the education group [40–43]. Therefore, goal setting and empowerment by self-efficacy can be defined as the key core for better health outcomes. In the present study, HC focused on self-empowerment and helped patients to set up health-related goals by using “Wheel of Health” which enabled patients to assess the interrelation between goals. Patients were followed up by coaching sessions where the motivation and empowerment to succeed the achievement were the key core. In the earlier study, we showed that patients who explored their self-management skills and competencies through HC sessions were more likely to attain healthy lifestyle; self-efficacy, physical activity, and toothbrushing shared one cluster. In the present study, satisfaction with the provider-patient communication was positively correlated with being physically active and regular toothbrushing in the HC group. Another way of describing, patients who were taking responsibility of their life by engaging in healthy lifestyles were more likely to be satisfied with their communication with their medical care providers. An explanation may be that HC, based on “learn-act-grow,” motivated patient to learn new information and to use his/her resources and then to act on behavioural change by unlocking his/her initial potential to grow continuously. This most probably led the patients in HC group to be actively involved in medical consultations, thereby learning and growing by taking the answers based on their own needs and expectations that led to an increased satisfaction. However, that requires further studies to explore the underlying pathways. There seems to be bilateral relationship between attaining healthy lifestyles and satisfaction with provider-patient communication. Therefore, it is important that medical professionals support patient motivation and help to empower engagement in healthy lifestyles, in particular considering the paradigm stating that patient satisfaction is a proxy measure for health [44, 45].

A limitation of the present study is the small sample size. Due to a number of organisational challenges, personnel, training, funding, time, and so forth, it was not possible to increase the number of participants. However, the original sample size is within the range of sample sizes of the studies in the field. Even though the sample is small and it is not representative of the general population of T2DM patients in Turkey, the study could be a model for further studies. Another limitation is that we did not measure the content of medical consultations to assess the differences between the HC and the HE groups; however, this was not in the scope of the intervention. Further studies need to explore the content and change in the content of medical/dental consultations. However, the study is, to our knowledge, the first of its kind which analyses the interrelation between patient's overall satisfaction with medical provider-patient communication on oral health and diabetes and psychobehavioural outcomes. The strengths of the study are, as discussed earlier by Cinar et al. [18], that (1) it has a comparison group (HC versus HE), (2) it has a relatively long period of intervention (16 months including a follow-up), (3) it is structured and uses internationally accredited content of HC, (4) it uses a validity-reliability tested self-efficacy measurement instrument, and (5) the health goals were set up by patients, thereby prioritizing the patients' own need and expectations. Furthermore, all HbA1c measures were taken from the records of the hospitals, so there was not any bias from self-reports.

## 5. Conclusion

The present study, for the first time to our knowledge, assessed that overall patient satisfaction with medical provider- (dentist- and physician-) patient communication was interrelated with oral health, diabetes, and psychobehavioural measures among T2DM patients, varying in different pathways in the HC and the HE groups. Our findings on the differentiation at pathways (clusterization) between HC and HE group can reveal that there is a threshold for patient satisfaction with medical provider-patient communication; above this threshold, personal measures such as self-efficacy and quality of life can be positively affected. HC in the present study significantly improved patient satisfaction with medical provider communication which was interrelated with health as a whole, in terms of clinical and psychobehavioural measures. HE improved satisfaction on communication with medical provider to a certain extent and that was interrelated only with clinical measures. The impact of HE does not seem to go deep or in another way of saying it cannot unlock the initial potential of the patient (self-competency); so the holistic interaction and improvement in clinical and personal measures seem to be lacking. There is a need for further studies to assess the impact of patient empowerment based HC on medical provider-patient communication, in particular on the content and interaction patterns between patient and the medical provider.

The results will hopefully encourage stakeholders and politicians to gain new insights for setting and supporting patient focused health promoting programmes like HC in primary health care settings especially for T2DM patients. This may facilitate dissemination, in order to improve both clinical and personal health outcomes at an individual and public health level, thereby helping to reduce the burden of T2DM. The HC methodology integrated programmes can significantly take part in reversing this epidemic.

## Figures and Tables

**Table 1:** The quality of life regarding daily diabetes management (modified WHOQOL-Bref).

To which extent or how much have you experienced certain things in the last 4 weeks?					
	Not at all	Little	A moderate amount	Very much	An extreme amount

To what extent do you feel that physical pain prevents you from doing what you need to do?					

How much do you need any medical treatment to function in your daily life?					

Do you have enough energy for everyday life?					

How much do you enjoy life?					

To what extent do you feel that your life is meaningful?					

How often do you feel hopeless, depressed, or anxious?					

**Table 2:** Between- and within-group differences from baseline to postintervention over sixteen months^*^.

	Health coaching group				Health education group				Between the groups (HC—HE)	
	*n*	Baseline	Postintervention	*P*	*n*	Baseline	Postintervention	*P*	*P*_baseline_	*P*_post-intervention _
HbA1c (%)	70	7.5%	6.9%	0.001	92	7.8%	7.8%	ns	ns	**0.001**
CPI	70	2.3 ± 0.86	0.6 ± 0.9	0.001	70	2.4 ± 1.23	2.4 ± 1.23	0.001	ns	**0.001**
Satisfaction on communication	69	6.3 ± 7.3	12,5 ± 4.04	0.008		7.3 ± 3.9	10.4 ± 4.6	0.001	ns	**0.006**
Physical activity (%)										
Physically inactive	77	42	15	0.001	65	46	48			
Physically active		55	69			48	42	ns	ns	**0.001**
Physically high active		4	16			6	10			
Toothbrushing (%)										
Never or rare	77	14	1	**0.001**	76	25	9			
2–5 times/week		20	11			23	22	**0.001**	ns	**0.001**
Once a day		34	18			30	44			
Twice a day		**32**	**70**			**22**	**25**			
TBSE (mean ± SD)	71	18,5 ± 11,9	29,3 ± 8,6	0.001	70	16,7 ± 11,8	20,9 ± 11,4	0.002	ns	**0.001**
Quality of life (mean ± SD)	68	13,5 ± 4,2	14,9 ± 4,5	0.003	65	13,9 ± 4,9	13,8 ± 4,6	ns	ns	ns

The total number for each variable differs because the same participants did not answer all the questions; *n* for each variable represents paired matches.

ns: nonsignificant.

^*^Based on the question with lowest rate of response (WHOQOL-Bref).

**Table:** (a) Health coaching group

	Quality of life
Satisfaction with provider-patient communication	0,693
CPI (periodontal treatment need)	−0,678
HbA1c	−0,610
Toothbrushing self-efficacy	0,449
Quality of life	0,760

The clusters in the study group, in total, accounted for 41.8% of the total variance. The cluster is named based on variable with the highest loading.

**Table:** (b) Health education group

	Oral health	Quality of life
Satisfaction with provider-patient communication	0,555	0,361
CPI (periodontal treatment need)	−0,765	∗
HbA1c	−0,717	0,527
Toothbrushing self-efficacy	∗	0,544
Quality of life	∗	0,840

The clusters in the study group, in total, accounted for 56.5% of the total variance [composed of component 1 (oral health): 30.7% and component 2 (quality of life): 25.8%].

^*^Loadings below 0.25 extracted for ease of communication. The clusters are named based on the variable with the highest loading.
